# Biopharmaceutical Characterization and Bioavailability Study of a Tetrazole Analog of Clofibric Acid in Rat

**DOI:** 10.3390/molecules22020282

**Published:** 2017-02-14

**Authors:** Nancy Vara-Gama, Adriana Valladares-Méndez, Gabriel Navarrete-Vazquez, Samuel Estrada-Soto, Luis Manuel. Orozco-Castellanos, Julio César Rivera-Leyva

**Affiliations:** 1 Facultad de Farmacia, Universidad Autónoma del Estado de Morelos, 62209 Cuernavaca, Morelos, Mexico; nvara83@yahoo.com.mx (N.V.-G.); vameadri25@hotmail.com (A.V.-M.); gabriel_navarrete@uaem.mx (G.N.-V.); enoch@uaem.mx (S.E.-S.); 2 Departamento de Farmacia, Universidad de Guanajuato, 36050 Guanajuato, Guanajuato, Mexico

**Keywords:** diabetes, bioavailability, clofibric acid, 11β-HSD1, tetrazole, dyslipidemia

## Abstract

In the current investigation, the physicochemical, biopharmaceutical and pharmacokinetic characterization of a new clofibric acid analog (Compound **1**) was evaluated. Compound **1** showed affinity by lipophilic phase in 1 to 5 pH interval, indicating that this compound would be absorbed favorably in duodenum or jejunum. Also, Compound **1** possess two ionic species, first above of pH 4.43 and, the second one is present over pH 6.08. The apparent permeability in everted sac rat intestine model was 8.73 × 10^−6^ cm/s in duodenum and 1.62 × 10^−5^ cm/s in jejunum, suggesting that Compound **1** has low permeability. Elimination constant after an oral administration of 50 μg/kg in Wistar rat was 1.81 h^−1^, absorption constant was 3.05 h^−1^, C_max_ was 3.57 μg/mL at 0.33 h, AUC_0–α_ was 956.54 μ/mL·h and distribution volume was 419.4 mL. To IV administration at the same dose, ke was 1.21 h^−1^, Vd was 399.6 mL and AUC_0–α_ was 747.81 μ/mL·h. No significant differences were observed between pharmacokinetic parameters at every administration route. Bioavailability evaluated was 10.4%. Compound **1** is metabolized to Compound **2** probably by enzymatic hydrolysis, and it showed a half-life of 9.24 h. With these properties, Compound **1** would be considered as a prodrug of Compound **2** with potential as an antidiabetic and anti dyslipidemic agent.

## 1. Introduction

### 1.1. Type 2 Diabetes (DMT2)

DMT2 is a chronic-degenerative disease characterized by a deficiency in the production of insulin by the β cells of the pancreas or the inability of the organism to efficiently use of insulin produces [1]. There were 6.4 million adults over the age of 20 diagnosed with diabetes in 2012 (National Health and Nutrition Survey) [2]. Due to this growing epidemiological trend, Diabetes Mellitus (DM) is considered a very significant public health problem in this country and around the world, and is one of the primary causes of death in the Mexican population.

**Figure 1:**
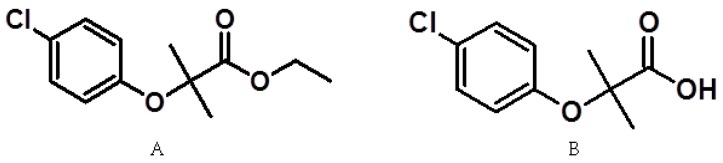
Chemical structure of clofibrate (**A**) and clofibric acid (**B**).

The complications associated with DMT2 include damage to the capillaries of the kidney, (causing glomerulonephritis and renal failure in its advanced stages), damage to the capillaries of the retina (causing blindness), peripheral neuropathy, myocardial infarction, cerebrovascular complications, and arteriosclerosis [3,4,5,6]. Some cases present dyslipidemia, a condition caused by alterations to the concentrations of lipoproteins in the blood. Overweight, obesity, physical inactivity, smoking, and alcohol consumption contribute to the development of this condition [6,7]. Treatments for dyslipidemia include inhibitors of the enzyme 3-hydroxy-3-methyl-glutaryl-coenzyme A reductase (statins), inhibitors of the absorption of cholesterol (ezetimibe), fatty acids such as omega-3, and fibric acid derivatives (bezafibrate, clofibrate and fenofibrate). Fibrates are prodrugs metabolized via enzymatic hydrolysis into active metabolites in the form of carboxylic acids. Clofibrate is metabolized (A) into clofibric acid (B, active metabolite) and is the drug of choice for the treatment of dyslipidemias associated with DMT2 [7,8]. Figure 1 shows the structure of the compounds described above. 

### 1.2. Pharmacological Activity of Fibrates

Clofibric acid is an agonist of nuclear receptors activated by peroxisome proliferator-activated receptor alpha (PPAR-α), whose function is related to processes that regulate the metabolism of fats, such as β-oxidation and adipogenesis [9,10]. PPAR-αs are significant therapeutic targets for the treatment of dyslipidemia. Fenofibrate has been reported to act as an anti dyslipidemic and has an inhibitory effect against the 11β-hydroxysteroid dehydrogenase type 1 (11β-HSD1), an enzyme expressed principally in muscle, liver and adipose tissue, which catalyzes the conversion of the inactive hormone cortisone to cortisol. This activates hepatic glycogenolysis, enabling glycogen to release glucose, thus further increasing glucose concentrations in the central circulatory system [11]. This enzyme is involved in the complications associated with DT2 [12,13].

**Figure 2:**
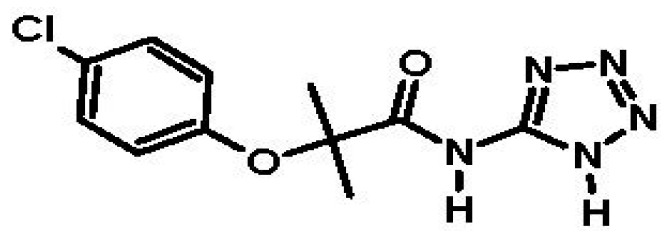
Chemical structure of 2-(4-chlorophenoxy)-2-methyl-*N*-(1*H*-tetrazol-5-yl) propanamide (tetrazole analog of clofibric acid) (Compound **1**).

Previous studies have shown that Saroglitazar (the PPAR α/γ dual agonist used for the treatment of DMT2 and dyslipidemia) produces a significant reduction in lipids and glucose in DT2 patients [14], while Chiglitazar (a PPAR α/γ dual agonist) reduces glucose levels and body weight in animals [15]. It is very possible that fibrates are able induce a double therapeutic effect, such as the abovementioned compounds, and control over both blood lipoproteins and hyperglycemia by inhibiting the hepatic glycogenolysis pathway activated by cortisol. The new compound 2-(4-chlorophenoxy)-2-methyl-*N*-(1*H*-tetrazol-5-yl) propanamide (Figure 2), a tetrazole isosteric analog of clofibric acid (Compound **1**), is an aza-heterocyclic amide derived from clofibric acid, to which a tetrazole ring (Log D of −0.37 and p*K*_a_ 4.9) was added as a surrogate of carboxylic acid (Log D of −1.65 and p*K*_a_ 4.76) [16]. The inhibitory effect of Compound **1** against the enzyme 11β-hydroxysteroid dehydrogenase type 1 (11β-HSD1) on the cellular line HEK293 (human embryonic kidney cells) was evaluated in vitro, demonstrating a moderate effect against the enzyme (51.17% inhibition at 10 μM) and a higher inhibition than clofibrate and clofibric acid [17,18]. The anti-diabetic effect of Compound **1** was determined at 50 mg/kg single dose using a non-insulin dependent diabetes mellitus rat model. The results indicated a significant reduction in glucose levels in the plasma, up to 77% 7 h post-administration, at which point it was compared with glibenclamide, which only reduced the glucose levels by 40% [18,19].

Given the potential of Compound **1** for the treatment of DMT2 and dyslipidemia, this study proposes the evaluation of its bioavailability in a rat model, as well as its biopharmaceutical characterization.

## 2. Materials and Methods

Dimethyl sulfoxide, *n*-octanol, mebendazole, Cremophor EL_pH-range__6.0–8.0_, naproxen, and amoxicillin were purchased from Sigma Aldrich (Steinheim, Germany). Methanol, acetonitrile, and water were HPLC grade, and the sodium hydroxide, phenolphthalein, monobasic sodium phosphate, phosphoric acid, and potassium biphthalate were obtained from J.T. Baker (J.T. Baker, Co., Center Valley, PA, USA). Clofibric acid (Compound **2**) was purchased from Sigma Aldrich (Steinheim, Germany). Compound **1** was synthesized in the Medicinal Chemistry Laboratory of the Pharmacy Faculty, Autonomous University of the State of Morelos (Universidad Autónoma del Estado de Morelos, or UAEM), Mexico. 

### 2.1. Physicochemical Evaluations

#### 2.1.1. p*K*_a_ Determination via Potentiometric Analysis

The determination of p*K*_a_ was undertaken as described by Babic A. et al. [18,20]. Compound **1** was prepared by weighing 0.001 equivalents, which were dissolved in the minimum volume of methanol, to which aqueous solution with an ionic strength of 0.02 M was added to obtain a final concentration for Compound **1** of 0.006 M. It was titrated with NaOH 0.01 N, which had been previously evaluated with an ionic strength of 0.02 M using Logger Pro 3.5.0 software (Vernier Software & Technology, Beaverton, OR, USA). The study was conducted in triplicate, determining the final points using the criteria from the first and second derivative test [21].

#### 2.1.2. Apparent Partition Coefficient

The octanol-water distribution coefficient was determined using the shake-flask method. The two phases were mutually saturated by shaking for 15 min. Phosphate buffers were used as the aqueous phase for the pH range 1.2 to 13. 5 mL portions of different buffer solutions (pH 1.2 to 13) containing 100 μg/mL of Compound **1** were mixed with 2.5 mL of *n*-Octanol. The mixture was shaken for 30 min, centrifuged at 8000× *g* for 10 min, after which the aqueous and organic phases were separated. Aqueous samples were analyzed at 222 nm using a validated spectrophotometric method (5–200 μg·mL^−1^) [22,23,24]. The distribution coefficient (log D) was calculated according to Equation (1) for each pH value:

(1) $$Log\;D=log\frac{\left[Compound\right]org}{\left[Compound\;\right]aq}$$

where *[Compound]_org_* is the drug concentration in the organic phase (determined by mass balance) and *[Compound]_aq_* is the drug concentration in the aqueous phase for each buffer solution. Every experiment was evaluated in triplicate. 

#### 2.1.3. Permeability Assay

Male Wistar rats weighing 200–250 g were used in this research. Everted intestinal sacs were prepared by quickly removing the small intestine from starved rats killed under ether anesthesia. All experiments were carried out after pre-incubation at 37 ± 0.5 °C, with the duodenum and jejunum then excised, flushed-through several times with saline solution at room temperature, and placed immediately into simulated intestinal fluid (SIF). The intestine was gently everted over a steel rod and filled with fresh SIF. The sacs were preincubated in oxygenated buffer solution for 5 min and then placed in 100 mL of oxygenated SIF solution containing 0.0001 M (100 μM) of Compound **1** at 37 °C. Oxygen was bubbled with a pump to maintain viable tissue [25]. 

The everted intestinal sac was filled with 1.5 mL of SIF solution, while a test compound solution (0.0001 M) remained external to the everted sac. The high and low permeability markers, naproxen and amoxicillin, were prepared in the same way [26]. 300 μL samples were collected from inside the sac with a metallic cannula at 5, 10, 15, 30, 45, 60, 90, and 120 min (apical-basolateral transport A-B), after which a medium replacement was made. Both an initial and final sample of the outside solution were analyzed by HPLC.

The apparent intestinal permeability (*P_app_*) was calculated according to Equation (2):

(2) $$Papp=\frac{V}{\left(A*Co\right)}dC/dt$$

where *P_app_* = Apparent Permeability (cm/s), *V* = Intestinal Volume (mL), *A* = Surface Area (cm^2^), *dC/dt* = Concentration Gradient, and, *Co* = Initial Concentration (μg/mL).

### 2.2. Chromatographic Conditions

The samples of Compound **1** were analyzed using a Waters chromatographic system equipped with a 717 plus auto-sampler, a model 515 isocratic pump, and a model 2487 UV-Vis detector. The software used was N2000 Chromatostation. The separation was performed on a 4.6 × 250 mm Zorbax CN (Agilent technologies, Santa Clara, CA, USA). The mobile phase consisted of phosphate buffer (0.05 M, pH 3.5) with 0.06% ethanol and methanol HPLC grade (64:36), filtered through 0.2 μm Nylon membrane (Nylaflo™) from PALL Corporation (Washington, NY, USA) and sonicated for 30 min. The flow rate was 1.0 mL/min. The column was kept at room temperature. Total run time was 25 min, with the detection carried out at 223 nm. The injection volume was 10 μL.

#### 2.2.1. Calibration Curve

A methanol solution of Compound **1** and clofibric acid (Compound **2**) was prepared to a final concentration of 100 μg/mL (working solutions). The calibration curves were prepared using appropriate volumes of Compound **1**, Compound **2**, and rat plasma to obtain the following concentrations: 0.46, 0.93, 3.75, 7.5, 15, 30 μg/mL (Compound **1**); and, 0.78, 1.56, 6.25, 12.5, 25, and 50 μg/mL (Compound **2**), simultaneously. Internal standard (mebendazole) was prepared at a concentration of 20 μg/mL (IS working solution).

#### 2.2.2. Sample Preparation

100 μL of plasma containing Compounds **1** and **2** at different concentrations were transferred to Eppendorf tubes, with 10 μL of IS working solution, 100 μL 1% solution phosphoric acid then added. Samples were shaken in a vortex for 30 s. Strata-X 33 μm cartridges (Polymeric Reversed Phase, 30 mg, Phenomenex) were conditioned with 2 mL of methanol and 1 mL of water, with the sample in the cartridge then left to stand for 2 min and immediately washed with 1 mL of a 95:5 water methanol solution. Finally, the sample was eluted four times with 500 μL of a 50:50 methanol acetonitrile solution. Samples were evaporated under atmospheric nitrogen at 40 °C. The waste reconstituted in methanol and 10 μL was injected into the HPLC.

### 2.3. Analytical Method Validation

The analytical method was validated in plasma using the following parameters: selectivity, linearity, precision, accuracy, absolute recovery, and LOQ [27].

#### 2.3.1. Selectivity

Blank plasma samples from different rat sources were prepared as previously described, to check for signals that might interfere with the detection of the analytes (Compound **1** and Compound **2**) or the IS. Additionally, the blank sample (a processed matrix sample without analyte and without IS) and a zero sample (a processed matrix with IS) were analyzed.

Also, specificity was demonstrated by comparing samples containing Cremophore EL and Heparine 10 UI/mL.

#### 2.3.2. Linearity 

The calibration curve was prepared in triplicate on two different days and the data analyzed as described above. The area ratio of Compound **1**/IS peaks (AR) was calculated. The linearity was determined between the 0.46–30 μg/mL recorded for Compound **1** and the 0.78–50 μg/mL recorded for Compound **2**, with the linear regression parameters as determined were the confidence intervals for slope and intercept.

#### 2.3.3. Precision and Accuracy

Aliquots of blank plasma were spiked with the corresponding volume of Compound **1** working solution and metabolite to obtain quality control (QC) samples containing 1.5 μg/mL (LQC), 18 μg/mL (MQC) and 24 μg/mL (HQC). For precision, the quality control samples were prepared in quintuplicate on two different days. Repeatability and reproducibility were calculated and expressed as RSD%. The within-run and between-run RSD% values were not to exceed 15% for each QC level. For accuracy, the QC samples were analyzed against a freshly calculated calibration curve, with the obtained concentrations compared with the nominal value. The accuracy was reported as a percentage of the nominal value. The mean concentration was to be within 15% of the nominal values for the QC samples.

#### 2.3.4. Extraction Efficiency

The efficiency of the extraction method was determined by comparing the plasma QC samples with aqueous QC solutions at corresponding levels for Compound **1** and Compound **2** to compare recovery, and measured in triplicate in the same analytical batch. Extraction efficiency was expressed as a percentage of the nominal concentration.

#### 2.3.5. Limit of Quantification (LOQ) and Limit of Detection (LOD)

The LOQ for the proposed methods was established through the analysis of the blank samples containing the lowest concentration level on each curve for every compound, determined experimentally by analyzing six replicates with suitable precision and accuracy (RSD < 20%).

## 3. Bioavailability Study

### 3.1. Animals

Male Wistar rats weighing 250–300 g were used for pharmacokinetic evaluation. Animals were individually identified by tail markings and acclimated to the study environment for seven days prior to the administration of the dose. Animals were kept on a 12 h light/dark cycle except when this was interrupted for study procedures. Animals were kept at average room temperature, which was regulated in the range of 18 to 29 °C. The animals were fasted prior to dose administration, and had access to water ad libitum.

### 3.2. Cannulation

Rats were anesthetized with ether and cannulated via the caudal vein using polyethylene tubing with an internal diameter of 0.023 inches, and an outer diameter of 0.038 inches (Becton Dickinson Clay Adams) [28].

### 3.3. Administration

Compound **1** was prepared with Cremophore (10%) in physiologic saline solution and administered orally in a 50 mg/kg dose, and via intravenous bolus (50 mg/kg). Study was conducted in quintuplicate.

### 3.4. Sample Collection 

After dosing, serial blood samples were collected (0.2 mL) 0, 0.083, 0.16, 0.25, 0.33, 0.50, 0.75, 1.0, 1.5, 2.0, 3.0, 4.0, 6.0, and 8 h after both oral and intravenous administration using heparinized (20 UI) tubes, and were centrifuged at 5000× *g* for 10 min, with the plasma harvested and analyzed by HPLC using the previously validated method.

## 4. Pharmacokinetic Analysis

The pharmacokinetic parameters of Compound **1** were obtained via non-compartmental analysis [29,30] using PK software (PKSolver). The STATA statistical program, version 12, was used for the comparison of the following: the pharmacokinetic parameters obtained via both administration pathways (C_max_ and T_max_); the elimination rate constant (k_el_); the biological half-life; the area under the curve (AUC_0–t_); the area under the curve from zero to infinity (AUC_0_–∞); volume of distribution (Vd); clearance (Cl); and, absolute bioavailability (F).

## 5. Bioethics Policies

This study followed the international recommendations for animal handling in biomedical research outlined in the Principles of Laboratory Animal Care (NIH publication #85-23, revised in 1985) and the current Mexican Official Standard NOM-062-ZOO-1999 on the management of laboratory animals [31,32].

## 6. Results and Discussion

### 6.1. Biopharmaceutical Properties

**Figure 3:**
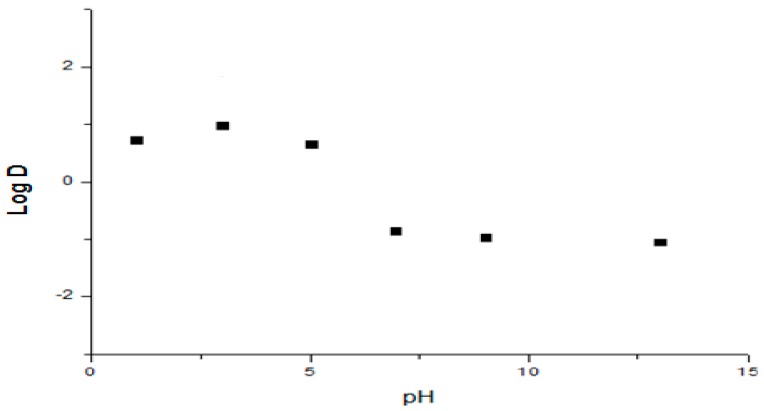
Log D of Compound **1** dependent on pH.

Compound **1** has a tetrazole ring and shows a good affinity for the lipophilic phase in the interval of pH evaluated from 1 to 5, in which the log D values obtained were positive (Figure 3). This indicates that Compound **1** could have a greater affinity in physiological intervals of pH from 1 to 5 in that non-ionized form prevails and is probably able to achieve a good rate of absorption at the stomach and duodenum section. At pH 7 Log D, the species with a higher level of ionization predominate, with the value recorded falling to below zero, indicating that Compound **1** has a greater affinity for the polar phase and a low affinity for the non-polar phase, reducing the probability of an improved absorption in the jejunum.

**Figure 4:**
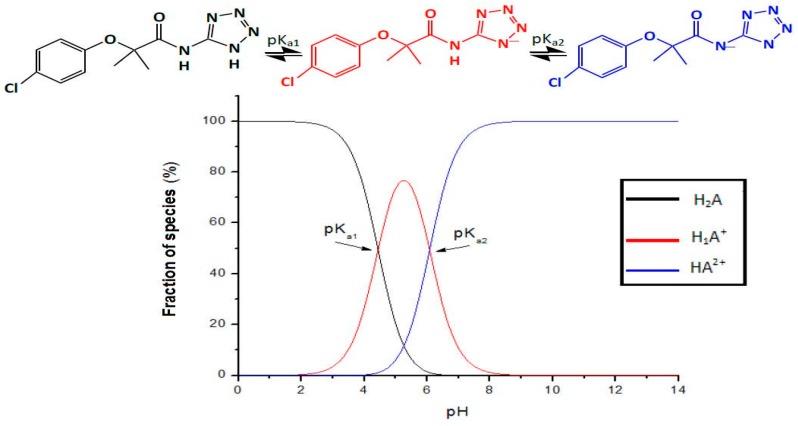
Dissociation equilibrium and speciation diagram for Compound **1**.

The p*K*_a_ values obtained (Table 1) suggest that the neutral form of the molecule of Compound **1** is found in the interval between pH 1 and 5, before the appearance of the first p*K*_a_ value, as observed in Figure 4.

As Figure 4 shows, the pH in the gastrointestinal tract at intervals from 1 to 8, Compound **1** could be non-ionized. Thus at a pH below 4, it could be capable of easily passing through the biological membranes via passive transport (transcellular transport). The dissociation equilibrium shows two ionized species of Compound **1** in the interval of physiological pH.

**Figure 5:**
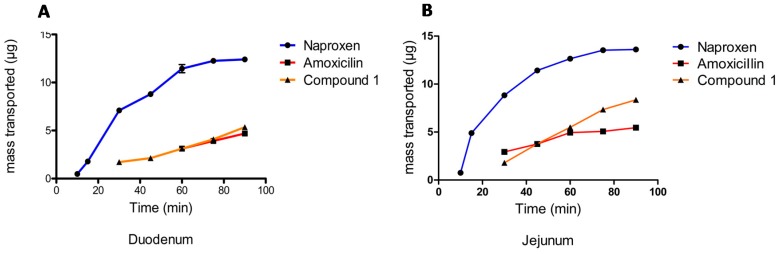
Permeability of Compound **1** in the everted rat gut model in the duodenum (**A**) and jejunum (**B**).

The evaluation of apparent permeability (*P_app_*) in the rat everted rat gut model (Figure 5), shows that Compound **1** has a coefficient of permeability similar to amoxicillin (the standard for low permeability used here) in both the duodenum and the jejunum. The values corresponding to the permeability of Compound **1** and the standards for high and low permeability are presented in Table 2. The permeability of Compound **1** is higher in the jejunum compared to the duodenum, with the standards of permeability showing the same behavior. However, Compound **1** shows a slightly higher level of permeability than the standard of low permeability (amoxicillin), thus possibly demonstrating better absorption than amoxicillin.

The levels of concentrations of Compound **1** were undetectable at the first few sampling times (5, 10 and 15 min), and were quantifiable between 30 and 90 min. While, at the same time, it was noted that the transport is slightly higher in the jejunum than the duodenum, compared to the standards (naproxen and amoxicillin), thus it could be considered a molecule with low permeability, as confirmed in the permeability results shown in Table 2. It should be noted that the *P_app_* value for clofibrate is −0.52 × 10^−6^ cm/s (pH 7.4) [33] and is higher than the permeability value for Compound **1** (ATAC) when compared to the value reported for clofibrate. Therefore, it can be deduced that Compound **1** is a molecule able to more easily and quickly permeate than clofibrate, thus improving its potential for absorption.

### 6.2. Validation of Analytical Method for Quantifying Compound ***1***

#### Chromatographic Conditions

**Figure 6:**
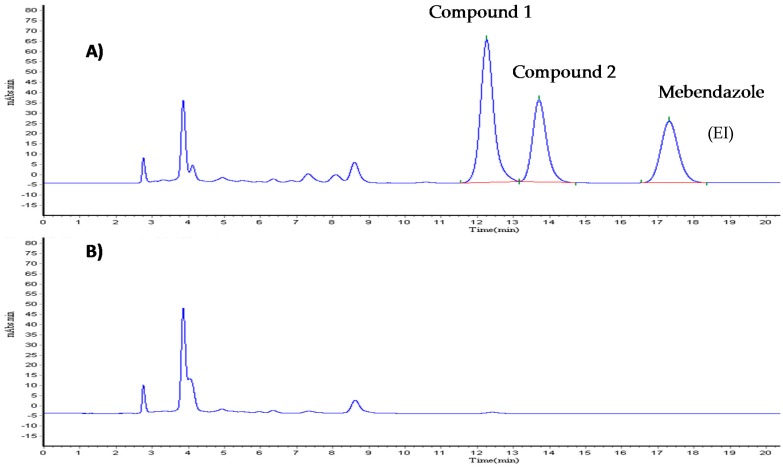
Chromatograms typical of Compound **1** and Compound **2** in rat plasma (**A**) and black of plasm (**B**).

The chromatographic system for Compound **1** in rat plasma showed the following: a resolution of 3.7 (Rs = 3.7) between Compound **1** and the internal standard (mebendazol); a resolution of 1.4 (Rs = 1.4) between Compound **1** and the Compound **2** (clofibric acid); an asymmetry of 1.2 for Compound **1**, 1.3 for the Compound **2** and 1.4 for the internal standard; and, a retention time of 13.3 min for Compound **1**, 11.9 for the metabolite, and 17.8 for the internal standard. Figure 6 shows a typical chromatogram for Compound **1** in rat plasma. The method was selective, in that there was no interference caused by endogenous compounds in the plasma or the products of degradation or impurities.

Table 3 shows the parameters of validation obtained for the method used for quantifying Compound **1**. The repeatability results for within-day-one variability present a percentage variation coefficient (RSD%) lower than 15%, while the reproducibility results for between-day variability present a RSD% lower than 15% in all the levels evaluated. The method used in this study demonstrated an absolute recuperation of over 98.0% across all levels of control, with the precision of the method ranging from 5.56% to 13.59% absolute percentage deviation.

The linearity of the method showed a correlation coefficient of over 0.99 in the interval of 0.46 to 30 mg/mL for Compound **1** and the 0.78 to 50 mg interval/mL for the Compound **2**. Table 4 shows the confidence intervals for the intercept and slope for both compounds. The limit of quantification was 0.46 mg/mL both for Compound **1** and Compound **2**. The limit detection was 0.30 mg/mL both Compound **1** and Compound **2**.

## 7. Bioavailability Evaluation

**Figure 7:**
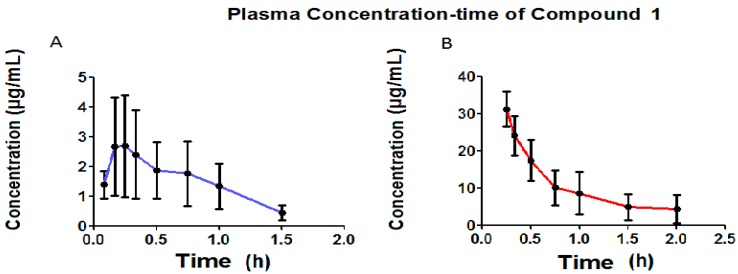
The Compound **1** plasma concentration (μg·mL^−1^) time profile obtained oral administration (**A**) and intravenous (**B**) in Wistar rats, mean ± SE (*n* = 6), respectively.

Figure 7 presents the pharmacokinetic profiles of Compound **1** after the oral and intravenous administration of a single 50 mg/kg dose. The non-compartmental analysis is shown in Table 5.

Figure 7 shows that, in orally administration, Compound **1** presents a C_max_ of 3.57 μg/mL, and that it was only possible to determine its plasmatic concentrations up to one hour after oral administration. Plasmatic concentrations were found up to two hours after intravenous administration.

Rapid absorption was observed for the oral administration of Compound **1**, presenting a constant average absorption of 3.05 h^−1^ and an average C_max_ of 3.57 μg/mL at 0.33 h. The average elimination rate constant for oral administration was 1.81 h^−1^, while, for intravenous administration this was 1.21 h^−1^, a difference which was not statistically significant using the *t*-test for unequal variances (*p* = 0.1410). The average distribution volume for oral administration is 419.4 mL, while this was 399.6 mL for intravenous administration, with neither administration pathways presenting a statistically significant difference (*p* = 0.8296). With the average distribution expressed in L/kg due to the fact that it varies along with the animal’s body weight, the calculation of the relationship between distribution volume and body weight gives an average distribution volume for oral administration of 1.43 L/kg and 1.30 L/kg for intravenous administration, indicating a wide distribution of Compound **1** in the rat organism. Rodents (rat) have been reported to present a total blood volume of 64 mL/kg, giving 3.2 mL [34] of total blood in a rat weighing approximately 200 g. This data shows that Compound **1** is widely distributed in the rat organism. Moreover, distribution volume is a constant parameter for a specific molecule. Compound **1** presents an average clearance (CL) of 751.2 mL/min for oral administration and 484.5 mL/min for intravenous administration, indicating a wide clearance in the rat organism. The clearance for Compound **1** for both administration pathways did not present statistically significant differences (*p* = 0.2018). The bioavailability of Compound **1** was 10.4%, which suggests that the metabolism suffers due to the effect of the first step. The average ABC_0–α_ for oral administration was 2.58 (μg·mL/h), which is lower than the average ABC_0–α_ recorded for intravenous administration, 38.8 (μg·mL/h), indicating that a lower amount of Compound **1** reaches the central circulatory system via oral administration. Some pharmacokinetic parameters for fibrates, such as clofibrate, which generates clofibric acid (its active metabolite) with an average lifetime of 7–8 h reported in the rat [35,36] are very different to Compound **1**, which presented an average lifetime of 0.71 h. It is likely that, in the future, Compound **1** could be used as a prodrug, in that it generates a metabolite with an average lifetime of 9 h, which is very similar to that reported for clofibric acid.

**Figure 8:**
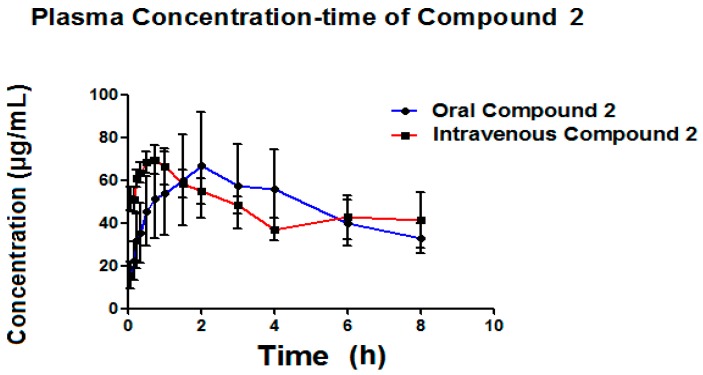
The Compound **2** plasma concentration (μg·mL^−1^) time profile obtained oral and intravenous in Wistar rats, mean ± SE (*n* = 6), respectively.

**Figure 9:**
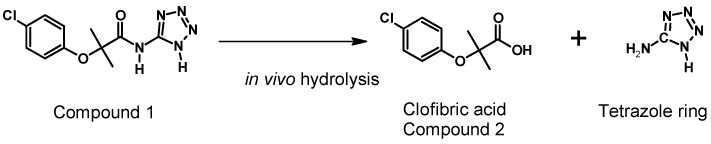
Hydrolysis reaction for Compound **1**.

Table 6 presents the pharmacokinetic parameters of the Compound **2** (metabolite) (Figure 8) formed by both administration pathways (oral and intravenous). This Compound **2** is possibly associated with clofibric acid through the administration of a single 50 mg/kg dose of Compound **1**, given that the metabolism of Compound **1** is thought to be principally related to enzymatic hydrolysis, as shown in Figure 9.

As seen in Figure 9, it was possible to detect, simultaneous to the analysis of Compound **1**, the appearance of a Compound **2**, which is possibly related to clofibric acid. 

Certain parameters of the metabolite were able to be detected (Table 6), such as: ABC_0–t_, ABC_0–α_, C_max_, and T_max_. It can be seen that the area below the curve for both metabolites is very similar, where, with intravenous administration, it can be seen that the metabolite is generated more quickly than with oral administration, indicating a higher plasmatic metabolism. The C_max_ for the metabolite obtained via intravenous administration is slightly higher (70.58 μg/mL) than the C_max_ obtained via oral administration (69.64 μg/mL), reinforcing the idea that Compound **1** is metabolized by the liver. The Compound **2**, as generated via both administration pathways, is capable of maintaining itself in concentrations still quantifiable after more than 8 h (Figure 8). Table 6 presents the pharmacokinetic parameters obtained for the Compound **2**. The elimination of constant of the Compound **2** (k_elm_) generated oral pathway is 0.10 h^−1^ on average, while for generated intravenous recorded an average of 0.11 h^−1^, results which do not present a statistically significant difference in the *t*-test (*p* = 0.1143). The average elimination of constant of the Compound **2** (k_elm_) generated via oral pathway (0.10 h^−1^) is very similar to that reported for clofibric acid in the rat (0.11 h^−1^) [36]. The average lifetime for the Compound **2** when generated oral pathway is 9.24 h and 9.0 h for the intravenous pathway, results which do not present a statistically significant difference in the *t*-test (0.1590). The average lifetime reported for clofibric acid administered orally in the rat is found to be within the range of 7–8 h [36], which provides evidence that the Compound **2** originated is likely to be said compound. The average ABC_0–t_ for the Compound **2** originating from the oral pathway was 340.94 (μ/mL·h), and 386.98 (μ/mL·h) for the intravenous pathway, results which were not statistically different, according to the T-test (*p* = 0.7317).

## 8. Conclusions

According to the results obtained, Compound **1** is a molecule of average polarity, with the non-ionized species formed having a pH lower than 5. It presents the behavior of a diacid and has a low permeability. However, its permeability is slightly higher than that shown by amoxicillin, the standard for low permeability. The average lifetime of its absorption was 3.05 h, while the elimination half-life was approximately 0.38 h, presenting a low level of bioavailability, with an average of 10.4%, generating a metabolite that is most likely to be clofibric acid. On appearing, said metabolite is responsible for the therapeutic effect, presenting an average elimination half-life of 9.24 h. This indicates that Compound **1** is a possible prodrug candidate, in that clofibrate is a liquid prodrug that is not very soluble in aqueous media and that generates its active metabolite, clofibric acid, via hydrolysis in vivo. This Compound **2** has a reported DL_50_ of 940 mg/kg [37] via oral administration in the rat, while, according to in silico predictions, Compound **1** has a DL_50_ of 2200 mg/kg [17] being a compound with a lower toxicity than clofibrate. It should also be noted that another advantage of Compound **1** is that it is a solid powder, making it easier to formulate than clofibrate. One of the disadvantages of Compound **1** is that, like clofibrate, it has little aqueous solubility, requiring it to be used in an adequate formulation in order to obtain optimal solubility for Compound **1** in aqueous media.

## Figures and Tables

**Table 1:** p*K*_a_ observed for Compound **1**.

Mean (±S.D.)
p*K*_a1_ 4.43 ± 0.10
*K*_a2_ 6.08 ± 0.14

**Table 2:** Permeability of Compound **1** in the everted rat gut model.

Compound	*P_app_* (cm/s)	
	Duodenum	Jejunum
Naproxen	3.02 × 10^−5^	3.98 × 10^−5^
Amoxicillin	8.52 × 10^−6^	1.00 × 10^−5^
ATAC	8.73 × 10^−6^	1.62 × 10^−5^

**Table 3:** Chromatographic parameters and validation results for the quantification method used for both Compound **1** and Compound **2**.

Compound	Validation Parameters					
	Compound 1			Compound 2		
Concentration (μg/mL)	(1.5)	(18 )	(24)	(1.5)	(18)	(24)
Within-day-one variability (RSD%)	5.73	14.72	5.06	8.16	3.26	1.52
Within-day-two variability (RSD%)	13.14	2.41	7.01	8.08	2.96	5.76
Between-day variability	10.21	10.42	6.27	7.76	7.89	4.53
Accuracy (%)	13.59	7.41	5.56	6.14	8.42	12.03
Absolute Recovery	109.4	95.01	95.23	105.3	106.5	108.9

**Table 4:** Linearity of quantitation method used for Compound **1** and the Compound **2** in rat plasma and confidence intervals (CI 95%) for slope and intercept.

Linearity						
	Compound 1 Confidence Intervals (α_0.05_)			Compound 2 Confidence Intervals (α_0.05_)		
	Mean Value (*n* = 4)	Lower	Upper	Mean Value (*n* = 4)	Lower	Upper
m	0.044	0.04279	0.05027	0.0273	0.02450	0.02903
b	−0.0087	−0.01881	0.01626	−0.0124	−0.05143	0.05516
r	0.9997			0.9989		
LOQ LOD	0.46 μg/mL 0.30 μg/mL					

**Table 5:** The mean pharmacokinetics parameters for Compound **1**.

Parameter	Oral Administration		Intravenous Administration	
	Mean	S.D.	Mean	S.D.
Kel (h^−1^)	1.81	0.09	1.21	0.59
Ka (h^−1^)	3.05	1.07		
T1/2el (h)	0.38	0.02	0.71	0.38
T1/2abs (h)	0.25	0.09		
C_max_ (μg/mL)	3.57	2.39		
T_max_ (h)	0.33	0.28		
ABC_0–t_ (μg/mL·h)	2.37	1.80	30.5	9.8
ABC_0–α_ (μg/mL·h)	2.58	1.97	38.8	19.2
Bioavailability (%)	10.4	0.04		
Cl (mL/h)	751.2	242.6	484.5	282.2
Vd (mL)	419.4	148.6	399.6	93.9
Vd (L/kg)	1.43	0.61	1.30	0.18
Dose (mg)	15.11	1.92	15.23	1.88

**Table 6:** The mean pharmacokinetics parameters for the Compound **2** generated both orally and intravenously.

Parameter	Oral Pathway		Intravenous Pathway	
	Mean	S.D.	Mean	S.D.
k_elm_ (h^−1^)	0.10	0.06	0.11	0.07
T_1/2__elm_ (h)	9.24	5.5	9.00	5.72
T_max_ (h)	2	0.71	0.75	0.29
C_max_ (μg/mL)	69.64	47.83	70.58	12.5
ABC_0–t_ (μ/mL·h)	340.94	135.77	386.98	243.57
ABC_0–α_ (μ/mL·h)	956.54	661.02	747.81	185.69
